# 靶向YAP/TAZ信号通路改善肿瘤免疫治疗的研究进展

**DOI:** 10.3779/j.issn.1009-3419.2025.102.08

**Published:** 2025-03-20

**Authors:** Pingxu ZHANG, Yiyi ZHAN

**Affiliations:** 830000 乌鲁木齐，新疆医科大学附属肿瘤医院; Tumor Hospital Affiliated to Xinjiang Medical University, Urumqi 830000, China

**Keywords:** YAP, TAZ, 免疫治疗, 免疫逃逸, 肿瘤微环境, YAP, TAZ, Immunotherapy, Immune evasion, Tumor microenvironment

## Abstract

尽管免疫检查点抑制剂（immune checkpoint inhibitors, ICIs）在肿瘤免疫治疗中取得突破性进展，但其疗效仍受限于免疫抑制性肿瘤微环境（tumor microenvironment, TME）。Yes相关蛋白（Yes-associated protein, YAP）和具有PDZ结合基序的转录共激活因子（transcriptional coactivator with PDZ-binding motif, TAZ）作为Hippo信号通路的关键效应因子，在肿瘤免疫逃逸中发挥关键作用。其可直接调控免疫检查点分子表达，介导免疫抑制微环境形成，抑制 T细胞功能，还与其他信号通路交互促进免疫逃逸。针对YAP/TAZ的抑制策略多样，包括直接靶向、调节上游机制和抑制下游靶基因等。临床前研究显示，YAP/TAZ抑制联合ICIs在多种肿瘤模型中疗效显著提升。本文就YAP/TAZ 在TME中影响免疫逃逸及靶向干预提升免疫治疗效果的研究进展进行综述，深入探讨基于该通路的联合治疗策略在转化医学中的潜在价值，以期为创新性免疫治疗方案的研发和临床精准化治疗决策提供理论框架与实践路径。

## Abstract (en)

Despite the groundbreaking advances in cancer immunotherapy achieved by immune checkpoint inhibitors (ICIs), their efficacy remains limited by the immunosuppressive tumor microenvironment (TME). Yes-associated protein (YAP) and transcriptional coactivator with PDZ-binding motif (TAZ), key effectors of the Hippo signaling pathway, play pivotal roles in tumor immune evasion. They directly regulate the expression of immune checkpoints, mediate the formation of an immunosuppressive microenvironment, inhibit T cell function, and interact with other signaling pathways to promote immune escape. Diverse strategies targeting YAP/TAZ have been developed, including direct inhibition, modulation of upstream regulators, and suppression of downstream target genes. Preclinical studies have demonstrated that combining YAP/TAZ inhibition with ICIs significantly enhances therapeutic efficacy across various tumor models. This review summarizes recent advances in understanding the role of YAP/TAZ in immune evasion within the TME and explores the potential of targeting this pathway to improve immunotherapy outcomes. Furthermore, it discusses the translational value of combination therapies based on YAP/TAZ inhibition, providing a theoretical framework and practical guidance for the development of innovative immunotherapeutic strategies and precision medicine approaches.

肿瘤免疫治疗作为继传统三大治疗手段（手术、放疗、化疗）后的第四大支柱治疗方法，其通过重编程机体免疫监视系统实现了从“直接杀伤”向“免疫调控”的治疗范式革新。以免疫检查点抑制剂（immune checkpoint inhibitors, ICIs）为代表的突破性疗法，通过靶向程序性细胞死亡受体1（programmed cell death protein 1, PD-1）、程序性死亡配体1（programmed death ligand 1, PD-L1）和细胞毒性T淋巴细胞相关蛋白4（cytotoxic T-lymphocyte-associated protein 4, CTLA-4）等关键免疫调控节点解除T细胞耗竭状态，在晚期实体瘤治疗中展现出独特的持久应答特征。临床转化研究^[1]^显示，纳武利尤单抗与帕博利珠单抗等代表性药物已推动黑色素瘤、非小细胞肺癌（non-small cell lung cancer, NSCLC）等肿瘤的5年生存率突破传统治疗瓶颈，其卓越疗效促使上述药物被纳入美国国立综合癌症网络（National Comprehensive Cancer Network, NCCN）、欧洲肿瘤内科学会（European Society for Medical Oncology, ESMO）等国际权威指南的优先推荐方案。ICIs的临床应用虽为晚期癌症治疗带来了革命性突破，但其疗效仍受限于肿瘤微环境（tumor microenvironment, TME）中复杂的免疫抑制网络。研究^[2⇓-4]^表明，TME中的异常血管生成、免疫抑制细胞浸润以及抗原呈递缺陷等机制，共同构成了免疫逃逸的复杂网络。

近年来，Hippo信号通路下游效应因子Yes相关蛋白（Yes-associated protein, YAP）和具有PDZ结合基序的转录共激活因子（transcriptional coactivator with PDZ-binding motif, TAZ）被证实为调控TME免疫抑制特性的关键枢纽。研究^[4⇓⇓-7]^表明，YAP/TAZ的异常激活通过多种机制促进免疫逃逸，包括上调PD-L1表达、招募免疫抑制性细胞群如调节性T细胞（regulatory T cells, Tregs）和髓源性抑制细胞（myeloid-derived suppressor cells, MDSCs）以及诱导基质纤维化，从而削弱ICIs的治疗效果。临床前研究^[8⇓-10]^进一步表明，靶向YAP/TAZ的干预策略能够显著增强ICIs的抗肿瘤活性，其机制涉及恢复T细胞功能、促进肿瘤血管正常化及增强抗原呈递等多个环节。然而，YAP/TAZ在肿瘤免疫调控中的时空异质性、组织特异性效应及其与其他信号通路的交互作用，仍是当前研究的难点和热点。因此，深入探讨YAP/TAZ抑制与提高肿瘤免疫检查点疗效的关系，不仅有助于揭示免疫治疗耐药的核心机制，还为开发基于YAP/TAZ靶向干预的联合治疗策略提供了理论依据和转化方向。本综述旨在系统总结YAP/TAZ调控肿瘤免疫的最新进展，评述其作为潜在治疗靶点的科学价值，并展望未来研究在精准医学中的应用前景。

## 1 YAP/TAZ概述

### 1.1 YAP/TAZ的分子结构特点与调控机制

YAP作为Hippo信号通路的核心转录共激活因子，其分子结构具有显著的功能模块化特征。该蛋白包含以下关键功能域：N端脯氨酸富集区、转录增强相关结构域（transcriptional enhanced associate domain, TEAD）、串联重复的双WW结构域、C端PDZ结合基序、SH3结合基序、卷曲螺旋结构域以及转录激活结构域^[11]^。值得注意的是，TAZ作为YAP的同源蛋白，虽然保留其核心功能结构域（包括TEAD结合域、单WW结构域、PDZ结合基序和转录激活域），但在进化过程中缺失了三个重要元件：N端脯氨酸富集区、第2个WW结构域以及SH3结合基序。这种结构差异可能导致二者在亚细胞定位、相互作用蛋白选择及下游信号调控等方面产生功能异质性。哺乳动物的Hippo信号通路主要发挥肿瘤抑制功能，其活性受到多级磷酸化激酶级联反应的精密调控。激酶级联主要包括哺乳动物Ste20样激酶1/2（mammalian Ste20-like kinase 1/2, MST1/2）、大肿瘤抑制激酶1/2（large tumor suppressor kinase 1/2, LATS1/2）、Salvador同源蛋白1（Salvador homolog 1, SAV1）和MOB激酶激活蛋白（MOB kinase activator, MOB）^[12]^。激酶链可以控制下游效应物YAP/TAZ的活化。活化的MST1/2与调节蛋白SAV1结合，进而促进LATS1/2和MOB的磷酸化，随后促进丝氨酸残基127处的YAP/TAZ的磷酸化^[13]^。这种磷酸化过程导致YAP/TAZ通过14-3-3蛋白相互作用在细胞质中滞留或者发生泛素化降解^[14]^。Hippo通路失活时，YAP/TAZ通过去磷酸化核转位，与TEAD形成转录复合体，激活细胞增殖相关基因及上皮间充质转化（epithelial-mesenchymal transition, EMT）核心程序，驱动促癌表型（图1）。Lamar等^[15]^研究表明，与TEAD家族结合是YAP发挥功能的重要步骤。

**图1:**
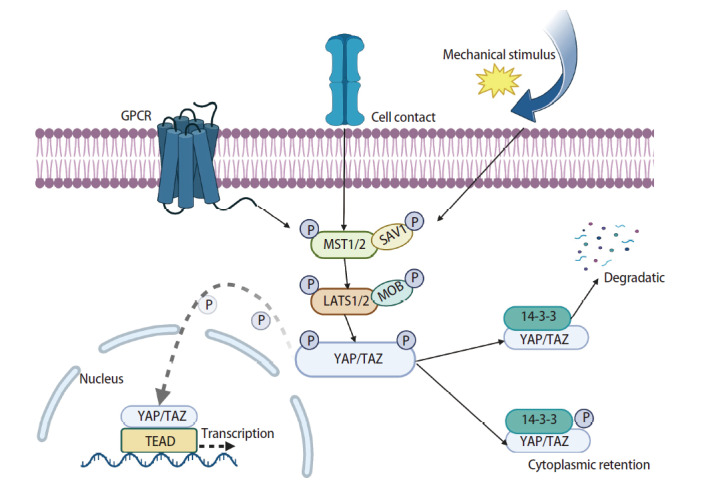
YAP/TAZ-TEAD信号轴的作用机制。细胞膜上的GPCRs、细胞间接触以及机械刺激可触发Hippo信号通路。Hippo信号通路核心MST1/2与SAV1、LATS1/2与MOB1分别形成的活性二聚体。通路激活时，LATS1/2使YAP/TAZ多位点磷酸化，磷酸化的YAP/TAZ与14-3-3结合，导致其滞留于细胞质并发生泛素化降解。通路关闭时，YAP/TAZ转移至细胞核，作为转录共激活因子与TEAD结合，启动促增殖和抗凋亡相关基因的转录程序。

### 1.2 YAP/TAZ的病理生理功能

YAP/TAZ主要是在细胞核中发挥作用，但他们也存在于细胞质中，通过亚细胞定位的动态变化发挥功能。长期激活YAP可导致肿瘤发生，许多癌症中都存在Hippo信号通路失调，表现为YAP/TAZ表达升高或核富集^[16]^。例如，在肺腺癌中，76%的病例显示YAP和TAZ的过度表达^[17]^。

YAP和TAZ在肺癌细胞中能够激活一系列与细胞增殖相关的基因，并且与不良预后、转移和治疗耐药性有关。在分子机制层面，YAP/TAZ-TEAD复合物通过特异性结合结缔组织生长因子（connective tissue growth factor, CTGF）、半胱氨酸丰富蛋白61（cysteine-rich protein 61, CYR61）等促增殖基因的启动子区域，激活细胞周期调控网络（如CDK4/6-Cyclin D轴），驱动肿瘤细胞恶性增殖^[18]^。同时，YAP和TAZ能够调节肺癌细胞的EMT过程^[19]^，形成复杂的调控网络。在EMT过程中，上皮细胞会失去细胞间黏附性和细胞极性，从而获得更强的迁移和侵袭能力。另外有研究^[20]^发现，YAP在肺癌干细胞（lung cancer stem cells, LCSCs）中过表达，通过激活相关基因表达来维持LCSCs特性。这种干性的维持使得肺癌细胞在接受常规治疗后能够存活下来，并且重新增殖，导致肿瘤复发。总之，YAP/YAZ主要通过促进细胞增殖、存活、迁移和侵袭，诱导EMT，促进基因组不稳定等促进肿瘤的发生发展^[19,21,22]^。因此，YAP/TAZ的异常激活在肿瘤的发病机制中逐渐凸显出其关键作用。

## 2 YAP/TAZ调控肿瘤免疫逃逸的分子机制

### 2.1 YAP/TAZ对免疫检查点分子的直接调控

PD-1是活化的T细胞表达的免疫检查点受体，作为一种重要的免疫抑制分子广泛参与免疫应答的调控。PD-L1作为PD-1的配体，在肿瘤细胞表面的PD-L1与活化的细胞毒性T细胞（cytotoxic T lymphocytes, TLs）表面PD-1结合后，从而特异性抑制T细胞受体（T cell receptors, TCRs）下游的磷脂酰肌醇3激酶-蛋白激酶B（phosphatidylinositol 3-kinase/protein kinase B, PI3K/Akt）和Ras-丝裂原活化蛋白激酶（Ras-mitogen-activated protein kinase, Ras-MAPK）信号级联反应。这种免疫检查点信号通路的异常激活不仅导致T细胞增殖阻滞及效应因子[如干扰素-γ（interferon-γ, IFN-γ）、颗粒酶B]分泌减少，更通过上调耗竭标志物T细胞免疫球蛋白和黏蛋白结构域-3（T-cell immunoglobulin and mucin-domain containing-3, TIM-3）、淋巴细胞活化基因-3（lymphocyte activation gene-3, LAG-3），诱导T细胞克隆失能，进而促进肿瘤的免疫逃逸^[23]^。研究^[5,24]^发现，YAP/TAZ可以通过转录调控的机制直接促进免疫检查点分子的表达。在转录水平上，YAP/TAZ能够与TEAD结合，形成YAP/TAZ-TEAD复合物，该复合物可直接结合到PD-L1基因的启动子区域，招募转录相关的辅助激活因子，从而促进PD-L1基因的转录。例如，在NSCLC中，YAP/TAZ与TEAD家族转录因子形成复合物后，可结合至PD-L1基因启动子区，显著上调其转录水平^[5]^，从而抑制T细胞功能（图2）。在肝细胞癌模型中，YAP通过激活TEAD-PD-L1轴使肿瘤细胞获得免疫逃逸能力，而YAP抑制可使PD-L1表达降低^[25]^。此外，YAP/TAZ能够通过激活PI3K-Akt信号通路，间接调节PD-L1的表达水平。Akt的磷酸化激活可促进下游转录因子如信号转导和转录激活因子3（signal transducer and activator of transcription 3, STAT3）或核因子κB（nuclear factor-kappa B, NF-κB）的核转位，这些因子通过特异性结合PD-L1基因启动子区的响应元件（如NF-κB位点或GAS序列），驱动其转录活性上调^[26,27]^。

**图2:**
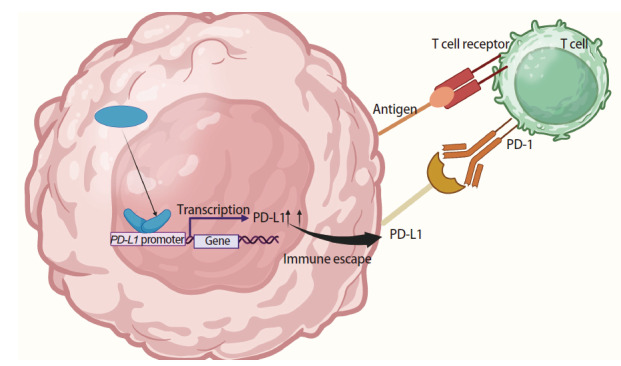
YAP在人类NSCLC中对PD-L1表达的调控机制。在NSCLC细胞内，YAP与TEAD结合，启动PD-L1基因转录，促使PD-L1表达上调。上调后的PD-L1被转运至 NSCLC细胞表面，与T细胞表面的PD-1结合，引发免疫逃逸。与此同时，T细胞通过其表面的TCRs识别NSCLC细胞呈递的抗原。

除了PD-L1，YAP/TAZ对其他免疫检查点分子也可能具有调控作用。对于CTLA-4，虽然目前具体机制尚不完全明确。现有的研究证据^[28]^显示，YAP/TAZ可能通过对表观遗传的重塑，间接地影响其自身的表达水平。例如，TAZ与组蛋白乙酰转移酶p300的相互作用可改变CTLA-4基因位点的染色质可及性^[29]^。在某些TME中，YAP/TAZ的高表达与CTLA-4表达的变化存在一定相关性^[30]^。LAG-3是一种重要的免疫检查点分子，它在肿瘤免疫逃逸的过程中扮演着关键角色。YAP/TAZ可能通过与特定的转录因子或信号通路相互作用，参与LAG-3的表达调控。例如，在黑色素瘤细胞中，初步研究^[31]^发现YAP/TAZ的异常激活可能导致 LAG-3表达上调。然而，关于这一过程的具体分子机制仍在研究中。

### 2.2 YAP/TAZ介导免疫抑制微环境形成

肿瘤免疫微环境是一个复杂的生态系统，YAP/TAZ通过促进免疫抑制细胞的浸润以及诱导肿瘤相关成纤维细胞活化和基质重塑，构建有利于肿瘤免疫逃逸的微环境。

Tregs和MDSCs是肿瘤免疫微环境中重要的免疫抑制细胞群体。YAP/TAZ可以通过多种方式促进这些细胞的浸润。一方面，YAP/TAZ激活后，肿瘤细胞分泌C-C基序趋化因子配体2（C-C motif chemokine ligand 2, CCL2）、CCL5等趋化因子，吸引MDSCs迁移至肿瘤组织，抑制T细胞功能，促进肿瘤进展^[4]^（图3）。在前列腺癌中^[3]^，YAP促使MDSCs分泌CXCL5，通过CXCL5-CXCR2信号通路，实现MDSCs在肿瘤部位的募集与大量积累，这些积聚的MDSCs抑制T细胞和自然杀伤（natural killer, NK）细胞功能，削弱机体免疫反应。此外，YAP/TAZ高表达的肿瘤还分泌CCL2，将肿瘤相关巨噬细胞（tumor-associated macrophages, TAMs）募集到TME，使其产生白细胞介素-6（interleukin-6, IL-6）和粒细胞-巨噬细胞集落刺激因子（granulocyte-macrophage colony-stimulating factor, GM-CSF），进一步增加MDSCs积累，抑制CD8^+ ^T细胞活性，营造免疫抑制性TME。另一方面，Tregs通过抑制效应T细胞功能，在肿瘤免疫中形成促癌作用。研究^[32]^显示，YAP依赖性信号激活可强化Tregs内转化生长因子-β（transforming growth factor β, TGF-β）/Smad通路，通过诱导Foxp3表达驱动其分化。进一步临床证据^[33]^表明，肝细胞癌患者外周血Tregs中YAP-1表达显著升高，其通过TEAD转录因子直接结合TGF-β受体II（TGF-β receptor II, TGFBR2）启动子，增强TGFBR2转录活性，最终形成促Treg分化及肿瘤免疫抑制微环境的正反馈环路^[34]^。

**图3:**
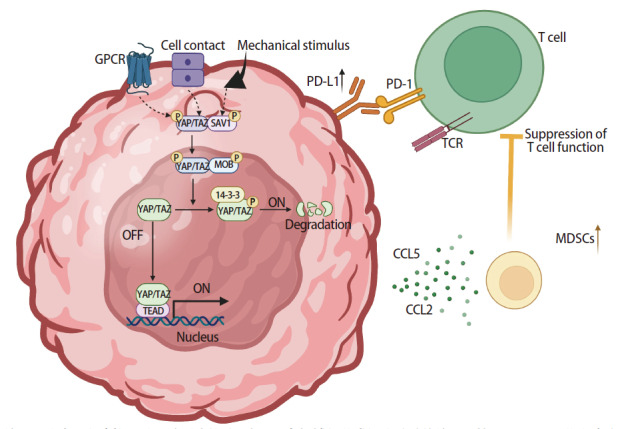
YAP/TAZ通过Hippo信号通路介导免疫抑制微环境形成的分子机制。在机械刺激或细胞接触等信号调控下，LATS1/2激酶磷酸化YAP/TAZ，促使其降解或核转位。核内YAP/TAZ与TEAD转录因子结合，驱动趋化因子CCL5和CCL2的表达，招募髓系抑制细胞等免疫抑制性细胞。此外，YAP/TAZ通过上调PD-L1等免疫检查点分子，协同抑制T细胞功能。该通路通过调控肿瘤微环境中的免疫抑制因子，促进免疫逃逸及微环境重塑。

### 2.3 YAP/TAZ对T细胞功能的抑制作用

T细胞为抗肿瘤免疫的核心效应细胞，CD4^+^和CD8^+^的活化、迁移及功能状态受YAP/TAZ通路严格调控。研究^[35]^表明，YAP通过调节TCR信号的阈值来限制T细胞活化。YAP缺陷型T细胞在抗原刺激下呈现激活标志物（CD44、CD25、CD69）的显著上调，并表现出更强的肿瘤归巢能力（如肺癌模型中CD8^+^ T细胞浸润增强）。此外，YAP/TAZ通过进行表观遗传重编程，显著影响T细胞的分化过程以及其耗竭进程：一方面，其可通过结合辅助性T细胞1（T helper type 1, Th1）/细胞毒性T细胞（cytotoxic T lymphocyte, CTL）分化相关转录因子（如T-bet）的启动子区，抑制效应T细胞功能成熟；另一方面，在慢性抗原刺激下，YAP/TAZ通过激活DNA甲基转移酶（DNA methyltransferases, DNMTs）和/或组蛋白去乙酰化酶（histone deacetylases, HDACs）等表观修饰酶，促进耗竭标志物（PD-1、TIM-3）的异常累积，加速T细胞功能失活^[36]^。这些机制共同导致抗肿瘤免疫应答强度降低，并削弱免疫治疗的临床响应。

临床前研究^[36]^证实，抑制YAP/TAZ可逆转T细胞功能抑制并增强免疫治疗效果：YAP/TAZ抑制剂不仅能提升T细胞增殖能力及效应分子（IFN-γ、Granzyme B）分泌水平，还可通过重塑表观遗传状态恢复耗竭T细胞的杀伤功能。更重要的是，联合ICIs可协同克服TME屏障，显著延长荷瘤模型生存期。这些发现提示，靶向YAP/TAZ通路为改善实体瘤免疫治疗抵抗提供了新策略，尤其针对T细胞浸润不足及T细胞耗竭主导型肿瘤微环境具有转化潜力^[37]^。

### 2.4 YAP/TAZ与其他信号通路的交互作用

YAP/TAZ并非孤立地发挥作用，其与其他信号通路之间存在广泛的交互作用，协同促进肿瘤免疫逃逸。Wnt/β-catenin信号通路在肿瘤的发生发展中起着重要作用，YAP/TAZ与Wnt/β-catenin通路之间存在密切的交叉对话。在某些情况下，YAP/TAZ可以与β-catenin相互作用，共同调节下游基因的表达。例如，在肝细胞癌中，YAP/TAZ和β-catenin可以同时激活，它们共同结合到一些与肿瘤免疫逃逸相关基因的启动子区域，促进这些基因的表达，从而增强肿瘤细胞的免疫逃逸能力^[38]^。此外，YAP/TAZ还与TGF-β信号通路相互影响。TGF-β是一种重要的免疫抑制细胞因子，YAP/TAZ 可以调节TGF-β信号通路的活性，反之亦然。在TME中，YAP/TAZ激活可以促进TGF-β的分泌，而TGF-β又可以通过激活下游的Smad信号通路，进一步增强YAP/TAZ的活性，形成一个正反馈调节环路，共同促进肿瘤免疫逃逸^[39]^。因此，深入研究这些信号通路之间的协同作用机制，对于揭示肿瘤免疫逃逸的本质，开发新的肿瘤免疫治疗策略具有重要意义。

## 3 YAP/TAZ抑制的策略与方法

在探讨YAP/TAZ抑制对提高肿瘤ICIs疗效的研究进展中，明确YAP/TAZ抑制的具体策略与方法是理解其作用机制及临床应用潜力的关键。YAP/TAZ作为Hippo信号通路的核心效应分子，其异常激活与肿瘤发生、发展及免疫逃逸密切相关。因此，针对YAP/TAZ的抑制策略不仅包括直接靶向其蛋白活性或表达的小分子抑制剂和基因编辑技术，还涉及间接调控其上游信号通路或下游效应分子的多种方法，因此鉴于YAP/TAZ与其他通路的协同作用，开发多靶点抑制策略成为重要方向。

### 3.1 直接靶向YAP/TAZ

作为首个被报道的YAP-TEAD小分子抑制剂，维替泊芬（Verteporfin）于2012年被证实可通过特异性干扰YAP-TEAD蛋白复合物的形成，显著抑制YAP依赖性肿瘤恶性表型^[40]^。近年来研究发现，维替泊芬在克服耐药性方面具有重要价值：其能够逆转肿瘤细胞对BRAF抑制剂^[41]^、拉帕替尼（Lapatinib）^[42]^及表皮生长因子受体（epidermal growth factor receptor, EGFR）抑制剂^[43]^等靶向药物的获得性耐药，这一作用与抑制YAP/TAZ-TEAD信号轴介导的旁路激活密切相关。值得注意的是，VGLL4作为内源性YAP-TEAD拮抗剂，可通过竞争性结合TEAD结构域发挥抑癌作用。基于此特性设计的VGLL4模拟肽（如Super-TDU）在临床前研究中展现出显著的抗肿瘤活性^[44]^，为开发新型YAP-TEAD靶向药物提供了新思路。进一步研究^[45]^发现，通过设计YAP样竞争性多肽可有效破坏YAP-TEAD复合物稳定性，进而抑制肿瘤进展。此外，针对TEAD棕榈酰化修饰的调控成为新兴策略——抑制TEAD自动棕榈酰化（如使用MGH-CP1化合物）可降低其与YAP的结合亲和力及转录活性，为靶向该通路提供了新方向^[46]^。

### 3.2 调节YAP/TAZ上游机制

甲羟戊酸通路作为调控YAP/TAZ活性的关键上游途径，其限速酶羟甲基戊二酰辅酶A（hydroxy methylglutaryl coenzyme A, HMG-CoA）还原酶的他汀类抑制剂（如辛伐他汀）可通过抑制GGTI/FTI介导的蛋白异戊烯化，阻断YAP/TAZ核转位并降低其转录活性^[47]^。临床研究^[24]^显示，NSCLC患者联用他汀类药物与EGFR抑制剂可显著提高总生存期，提示靶向代谢通路可能成为克服YAP相关耐药的有效策略。此外，细胞力学信号传导通路（如FAK-SRC-PI3K和ROCK通路）对YAP/TAZ的调控作用已被阐明：FAK抑制剂VS-4718可通过阻断SRC磷酸化抑制YAP核聚集^[48]^，而ROCK抑制剂法舒地尔（Fasudil）则通过破坏细胞骨架张力来降低TAZ稳定性^[49]^。值得关注的是，达沙替尼通过抑制SRC激酶活性阻断YAP活化，并协同抑制MEK/ERK通路，从而增强KRAS突变肺癌对曲美替尼的敏感性^[48,50]^。此外，其与EZH2抑制剂联用可协同靶向Hippo通路和表观遗传调控^[51]^，充分展现了跨信号网络联动的治疗潜力。

### 3.3 抑制YAP/TAZ的下游靶基因

针对YAP/TAZ下游靶基因的精准干预已成为逆转治疗抵抗的重要策略。研究^[52]^显示，在BRAF或MEK抑制剂耐药模型中，YAP/TAZ可通过转录上调抗凋亡蛋白BCL-xL促进肿瘤细胞存活，而联用BCL-xL抑制剂ABT-263可使耐药细胞恢复药物敏感性[半抑制浓度（half inhibitory concentration, IC_50_）降低5-8倍]。在EGFR抑制剂耐药的NSCLC中，YAP介导的AXL过表达被证实是关键耐药机制，使用选择性AXL抑制剂TP-0903可显著增强奥希替尼的疗效^[53]^。然而，YAP/TAZ的转录调控网络具有显著肿瘤异质性，例如在肝癌中主要激活CTGF促纤维化程序^[54]^，而在乳腺癌中则优先调控ANKRD1等EMT相关基因^[55]^。因此，开发基于分子分型的下游靶点选择策略（如单细胞测序指导的靶向干预）将成为未来研究重点。

## 4 基于YAP/TAZ抑制联合ICIs

随着癌症研究的不断深入，YAP/TAZ信号通路作为癌症治疗关键靶点的重要性日益凸显。该通路不仅在肿瘤细胞的增殖、迁移和侵袭中发挥核心作用，还通过调控TME中的免疫细胞功能，促进免疫逃逸和耐药性的形成，与多种癌症的进展和不良预后密切相关。近年来，随着ICIs在癌症治疗领域取得突破性进展，研究者逐渐认识到YAP/TAZ通路的抑制可能增强免疫治疗的疗效。因此，YAP/TAZ抑制剂与免疫疗法的联合应用成为癌症治疗领域的热点研究方向，其潜在协同效应备受关注。目前，多项临床试验正在积极推进，并已取得了一些令人鼓舞的初步结果。

在多种肿瘤动物模型中，YAP/TAZ抑制联合免疫检查点阻断展现出良好效果。在结肠癌小鼠模型中，Zhuang教授团队^[9]^开发了一种基于维替泊芬（YAP1抑制剂）、电离辐射和抗PD-1抗体的三联疗法。该疗法通过YAP1抑制诱导免疫原性细胞死亡，联合放疗增强肿瘤抗原释放，并协同PD-1阻断激活抗肿瘤免疫反应，实验数据显示，联合治疗组中约40%的小鼠实现肿瘤完全消退，60%的肿瘤体积缩小超过50%。Gao教授团队^[10]^揭示了YAP1调控肝癌自噬的新机制及其免疫治疗协同作用。研究发现肝癌组织中YAP1与自噬标志物LC3B表达呈正相关（r=0.31, P<0.001）。同时抗PD-1治疗会激活YAP1通路并提升LC3B表达，提示其参与免疫耐药。通过联合YAP1敲除与抗PD-1治疗，可使小鼠肿瘤负荷降低，表明联合抗PD-1治疗和YAP1敲除可抑制自噬、增强抗肿瘤效果。在针对原发性胆管癌的实验中，Fu教授团队^[8]^开展了深入的体外研究。结果显示，抗PD-1与维替泊芬联合治疗组的肿瘤大小以及终点测量的肿瘤重量，均小于单药治疗组，这一数据差异充分体现出二者联合具有协同抗肿瘤效应。进一步对肿瘤浸润淋巴细胞（tumor-infiltrating lymphocytes, TILs）进行分析发现，联合治疗组中CD4^+^ T细胞和NK细胞的百分比呈现出增加趋势，然而其他TILs子集数量变化并不显著。这一现象表明，维替泊芬或许能够对肿瘤TME起到调节作用，进而增强ICIs在胆管癌治疗中的抗肿瘤活性。

肿瘤的发生发展涉及多个信号通路的协同异常激活，单一通路抑制往往难以全面阻断肿瘤的恶性进程。研究^[38,39]^表明，除了抑制YAP/TAZ通路外，联合靶向Wnt/β-catenin、TGF-β等与肿瘤增殖、侵袭和转移密切相关的通路，有望实现对肿瘤细胞更全面的抑制。通过同时阻断多条关键信号通路，可以干扰肿瘤细胞的生存和发展机制，降低肿瘤细胞的耐药性，从而提高治疗效果。临床前研究^[56]^已证实，Tankyrase抑制剂G007-LK通过双重抑制Wnt/β-catenin和YAP通路，显著增强抗PD-1疗效。例如，在β-catenin高表达的B16-F10黑色素瘤模型中，G007-LK联合抗PD-1治疗使肿瘤体积减少83%，18.5%的小鼠实现肿瘤完全消退，且这一疗效依赖于β-catenin的敲除以及CD8^+ ^T细胞和IFN-γ的激活。因此，联合靶向Wnt/β-catenin、YAP/TAZ及TGF-β等通路，可以通过多维度破坏肿瘤增殖-转移-免疫逃逸网络，降低耐药风险并增强治疗效果，为实体瘤治疗提供新的策略。

## 5 总结与展望

YAP/TAZ作为Hippo信号通路的核心效应因子，通过调控PD-L1表达增强其转录、介导免疫抑制微环境形成并促进MDSCs/TAMs募集及Tregs分化以及与Wnt/β-catenin、TGF-β等信号通路的交互作用，成为肿瘤免疫逃逸的关键驱动因素。

研究^[40,46]^表明，靶向YAP/TAZ的小分子抑制剂（如维替泊芬）、TEAD棕榈酰化调控策略等可通过阻断YAP-TEAD复合物形成或抑制其核转位，有效逆转免疫抑制微环境，增强T细胞抗肿瘤活性。临床前研究^[9,10]^进一步证实，YAP/TAZ抑制联合ICIs在结肠癌、肝癌等模型中显著提升疗效，为克服ICIs耐药性提供了新思路。

然而，YAP/TAZ信号在肿瘤免疫调控中呈现复杂双向性：其抑制虽可解除免疫抑制并增强ICIs疗效，但也可能因干扰T细胞功能或组织特异性效应而限制临床应用^[36]^。因此其临床转化仍面临诸多挑战。首先，YAP/TAZ在正常组织中广泛表达并参与多种生理过程（如组织再生、细胞增殖和分化），其抑制可能导致严重的脱靶效应和组织特异性毒性^[57]^。例如，YAP/TAZ在肝脏再生和心脏发育中的关键作用可能限制其抑制剂在肝癌和心血管疾病患者中的应用^[58,59]^。其次，现有YAP/TAZ抑制剂（如维替泊芬）的选择性和药代动力学特性仍需优化，以避免对正常组织的非特异性损伤。

未来研究需进一步明确YAP/TAZ在肿瘤免疫调控中的时空异质性和组织特异性效应，深入探究其与其他信号通路复杂的交互作用机制，尤其是在不同肿瘤类型和疾病发展阶段的差异，这将为精准治疗提供更坚实的理论基础。此外，需结合临床队列数据，系统验证YAP/TAZ抑制与ICIs、靶向Wnt/TGF-β通路药物的协同效应，建立跨组学生物标志物（如PD-L1/YAP共表达谱）预测模型，指导患者分层与精准联用策略；最后，可以通过多中心随机对照试验，评估联合治疗的长期生存获益及耐药复发风险，同时开发动态监测技术[如循环肿瘤DNA（circulating tumor DNA, ctDNA）-YAP活性追踪]，实时优化临床干预策略。

综上，靶向YAP/TAZ联合免疫检查点阻断的治疗策略，有望通过重塑肿瘤免疫微环境突破现有疗效瓶颈。未来的研究应集中于针对YAP/TAZ的联合治疗策略，并进行更多相关的动物模型和临床试验，以深入探讨这种策略在免疫治疗中的有效性和安全性。这将为克服免疫治疗耐药性以及提升免疫治疗效果开辟新的路径。同时，还需要进一步梳理Hippo通路各个组分在肿瘤免疫微环境中的复杂调控网络及其分子机制。这将有助于揭示更多潜在的治疗靶点和生物标志物，并将为肿瘤患者提供更高效、个性化的治疗选择，推动肿瘤免疫治疗领域的进步与发展。
